# Changes in lower dental arch dimensions and tooth alignment in young adults without orthodontic treatment

**DOI:** 10.1590/2176-9451.20.3.064-068.oar

**Published:** 2015

**Authors:** Bruno Aldo Mauad, Robson Costa Silva, Mônica Lídia Santos de Castro Aragón, Luana Farias Pontes, Newton Guerreiro da Silva, David Normando

**Affiliations:** 1 Specialist in Orthodontics, ABO-PA, Belém, Pará, Brazil; 2 MSc in Orthodontics, Universidade Federal do Pará (UFPA), School of Dentistry, Belém, Pará, Brazil; 3 Adjunct professor, Universidade Federal do Pará (UFPA), School of Dentistry, Belém, Pará, Brazil; 4 Adjunct professor, Universidade Federal do Pará (UFPA), School of Dentistry, Belém, Pará, Brazil. Coordinator, Universidade Federal do Pará (UFPA), postgraduate program in Dentistry. Coordinator, ABO-PA, specialization program in Orthodontics. Belém, Pará, Brazil

**Keywords:** Malocclusion, Dental arch, Mandible, Maxillofacial development, Dental crowding

## Abstract

**OBJECTIVE:**

The aim of this longitudinal study, comprising young adults without orthodontic treatment, was to assess spontaneous changes in lower dental arch alignment and dimensions.

**METHODS:**

Twenty pairs of dental casts of the lower arch, obtained at different time intervals, were compared. Dental casts obtained at T_1_ (mean age = 20.25) and T_2_ (mean age = 31.2) were compared by means of paired t-test (p < 0.05).

**RESULTS:**

There was significant reduction in arch dimensions: 0.43 mm for intercanine (p = 0.0089) and intermolar (p = 0.022) widths, and 1.28 mm for diagonal arch length (p < 0.001). There was a mild increase of approximately 1 mm in the irregularity index used to assess anterior alignment (p < 0.001). However, regression analysis showed that changes in the irregularity index revealed no statistically significant association with changes in the dental arch dimensions (p > 0.05). Furthermore, incisors irregularity at T_2_ could not be predicted due to the severity of this variable at T_1_ (p = 0.5051).

**CONCLUSION:**

Findings suggest that post-growth maturation of the lower dental arch leads to a reduction of dental arch dimensions as well as to a mild, yet significant, increase in dental crowding, even in individuals without orthodontic treatment. Furthermore, dental alignment in the third decade of life cannot be predicted based on the severity of dental crowding at the end of the second decade of life.

## Abstract (pt)

**OBJETIVO:**

o objetivo deste trabalho foi avaliar, por meio de um estudo longitudinal em adultos jovens, sem tratamento ortodôntico, as alterações espontâneas do alinhamento da arcada dentária inferior e de suas dimensões.

**MÉTODOS:**

vinte pares de modelos de gesso da arcada inferior foram obtidos em dois tempos. No primeiro exame (T_1_), os indivíduos tinham, em média, 20,25 anos; enquanto no segundo exame (T_2_) a média de idade foi de 31,2 anos. Comparações entre os tempos T_1_ e T_2_ foram realizadas usando o teste *t* pareado (p < 0,05).

**RESULTADOS:**

houve uma redução significativa nas dimensões da arcada - de 0,43mm nas larguras intercaninos (p = 0,0089) e intermolares (p = 0,022) e de 1,28mm para o comprimento diagonal da arcada (p < 0,001). Foi observado um aumento suave, de aproximadamente 1mm, no índice de irregularidade anterior (p < 0,001). Entretanto, a análise de regressão mostrou que as mudanças no índice de irregularidade não revelaram uma associação estatisticamente significativa com as mudanças na arcada dentária (p > 0,05). Além disso, o índice de irregularidade dos incisivos em T_2_ não pode ser estimado, devido à severidade dessa variável em T_1_ (p = 0,5051).

**CONCLUSÃO:**

esses achados sugerem que a maturação da arcada dentária inferior, pós-crescimento, leva a uma redução das dimensões da arcada e um aumento suave, porém significativo, do apinhamento dentário, mesmo em indivíduos sem tratamento ortodôntico. Assim, o alinhamento dentário na terceira década de vida não pode ser previsto tendo como base a severidade do apinhamento dentário ao final da segunda década de vida.

## INTRODUCTION

Changes in the dental arch should be assessed to identify etiological factors associated with tertiary crowding, a phase when there is an increase in the demand of adult patients for orthodontic treatment or retreatment.

Numerous longitudinal studies have been conducted to assess changes in dental arch aligment,^1^^-^ 5 especially in the lower arch where the most significant post-treatment changes occur.^6^Changes in the first two decades of life have already been widely studied;^1^^,^ 4 ^,^ 7 ^,^ 8 however, there is lack of knowledge about occlusal changes in adults, particularly spontaneous changes in subjects without orthodontic treatment.

The literature shows different opinions about the etiology of lower incisors crowding, considering reduction in arch perimeter as the main causal factor. Other authors also point out the presence of a third molar^9^ or multiple factors.^4^

As regards adults, clinical studies often report orthodontically treated cases,^10^^-^ 13 thereby hindering evaluation of physiological changes in dental alignment. Thus, analysis of untreated cases is an excellent opportunity to determine the physiological changes in dental alignment, in addition to being the key to plan the retention phase and the removal timing for retainers.

This study aims to assess spontaneous changes in lower dental arch dimensions and incisors alignment by means of a longitudinal investigation conducted with young adults without previous orthodontic treatment.

## MATERIAL AND METHODS

This study was approved by Universidade Federal do Pará Institutional Review Board (CEP-ICS/UFPA) under protocol #366.357/2013.

The sample comprised 20 young adults (9 males and 11 females ) with mean age of 20.25 years old (15-24 years). Their records were retrieved from a previous study^14^ assessing 40 subjects, most of which were undergraduate students. Eleven years later (T_2_), 20 individuals with a mean age of 31.75 years were reassessed. Most subjects who were not reassessed at T_2_ had moved away. Therefore, the final sample was smaller. Dental casts were obtained for all subjects at T_1_ and T_2_ (n = 20). Only the lower dental arch was measured.

In selecting the sample, the following inclusion criteria were applied: no missing teeth or history of extractions in the lower arch, and no orthodontic or prosthodontic treatment carried out during the observation period.

**Figure 1.**
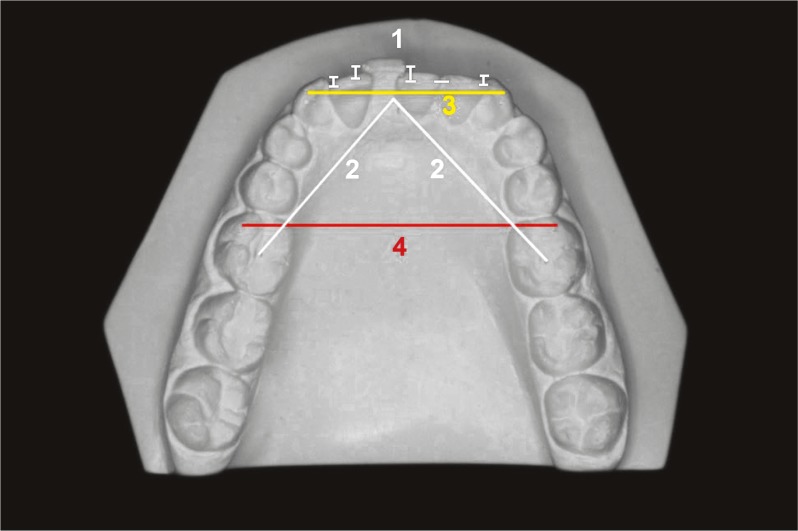
Irregularity index (1), arch length (2), intercanine width (3) and intermolar width (4).

All measurements (Fig 1) were performed by a single previously calibrated operator, and repeated after a three-day interval by means of a digital caliper (Utustools/Concepción, Chile) with precision of 0.02 mm. Whenever first and second measurements differed in more than 0.3 mm, they were retaken. Mean was used for statistical analysis.

The following measurements were investigated: Little's irregularity index,^15^ dental arch length, intercanine and intermolar widths (Fig 1). Little's irregularity index was the sum, expressed in millimeters, of the linear displacements of five contact points in lower incisors region. Arch length was obtained as the distance from lower inter-incisors region, at the level of palatal papilla, to the center of the first molar on both sides. Intercanine width was measured as the maximum distance between the cusp tips of lower canines; and intermolar width was measured as the greatest distance between the mesiobuccal cusp tips of lower first permanent molars.

Statistical analyses were performed by means of BioEstat 5.3 software (Mamirauá Institute, Belém, Pará, Brazil). Data were submitted to analysis of normal distribution by D’Agostino-Pearson test. T_1_ and T_2_ were compared by means of paired t-test at p < 0.05. Additionally, multiple regression analysis investigated whether increase in lower incisors irregularities could be predicted on the basis of initial data (T_1_).

## RESULTS

Data had normal distribution; therefore, to assess the differences between T_1_and T_2_, paired t-test was used.

From T_1_ to T_2_, there was an increase of 1.01 mm in Little's index (p < 0.0001). All other measurements were smaller at T_2_ (Table 1): arch length on the right side (-0.51 mm, p < 0.001) and left side (-0.77 mm, p < 0.0001), intercanine width (-0.43 mm, p < 0.0089) and intermolar width (-0.43 mm, p < 0 .0022).

**Table 1.** Mean, standard deviation (SD) and p value for each measurement at T1 (initial examination) and T2 (11.5 years later).

	T_1_	T_2_	T_2_ – T_1_	p value
	Mean (SD)	Mean (SD)	X dif	(paired t-test)
LI	4.64 (1.81)	5.65 (2.00)	1.01	<0.0001**
RHL	32.37 (1.86)	31.86 (1.88)	-0.51	<0.001**
LHL	32.47 (1.93)	31.70 (2.09)	-0.77	<0.0001**
IcW	25.55 (1.52)	25.12 (1.74)	-0.43	0.0089**
ImW	44.83 (2.72)	44.40 (2.87)	-0.43	0.0022**

LI = Little's index; RHL = Right hemiarch length; LHL = Left hemiarch length; IcW = Intercanine width; ImW = Intermolar width.

Regression analysis revealed that none of the independent variables were statistically associated with changes observed in Little's index (Table 2).

**Table 2.** Regression analysis of changes in Little's index at T2-T1 (dependent variable) and the following independent variables: intercanine width (IcW), intermolar width (ImW), Little's index (LI) at T1 and total arch length (TAL).

Independent variables	IcW T_1_		ImW T_1_		LI T_1_		TAL		F	p value
Dependent variable	R^2^	p value	R^2^	p value	R^2^	p value	R^2^	p value		
LI T_2_-T_1_	0.73	0.3179	14.5	0.0869	0.01	0.1938	10.35	0.1636	1.29	0.3179

## DISCUSSION

Dental arches are subjected to dimensional changes throughout life.^5^^,^ 11 ^,^ 16 ^,^ 17 Many studies have investigated orthodontic treatment stability by assessing the morphological changes of dental arch after the retention phase.^17^^-^ 20 These changes justify relapse of dental crowding in the lower dental arch. However, it is challenge to determine whether these dimensional changes would be a relapse of orthodontic treatment or a result of dental arch maturation. Identifying risk factors that could predict these changes is also important.

There is some evidence showing that the morphological and dimensional changes observed in dental arches after orthodontic treatment also occur in individuals who were not submitted to it.^3^^,^ 4 ^,^ 6 ^,^ 16 ^,^ 20 Nevertheless, in these cases, changes occur on a smaller degree than they do in samples of orthodontically treated cases.^4^ These studies assessed changes occurring in the second decade of life. Changes after this age have been poorly investigated.

The presence of third molars and their influence on crowding provokes controversy among dentists.^9^ Some studies identified a positive relationship between third molars and this malocclusion;^5^ however, several studies have refuted the hypothesis that the third molar exerts influence over crowding.^21^ Therefore, this issue did not receive major attention in the present study.

Our findings showed that the mean increase in lower incisors irregularity, from the age of 20 to 30 years old, was 1.01 mm (p < 0.0001). However, 55% of subjects showed less than 1 mm increase in the irregularity index, and only one patient had more than 3 mm of dental crowding. These findings are similar to those found in previous research, in which there were no reports of severe degrees of lower incisors irregularity.^3^^,^ 4 ^,^ 6 ^,^ 22

As regards lower dental arch morphology, intercanine width showed a reduction greater than 0.3 mm in 11 out of 20 subjects, with a mean of 0.43 mm (p < 0.001), thereby corroborating the results of previous studies^4^^,^ 6 ^,^ 23 ^,^ 24 examining other age groups. Sinclair and Little^4^ found a decrease in intercanine width of 0.44 mm from 13-14 years to 19-20 years, whereas Bishara et al^24^ found a decrease of 0.5 mm for male subjects and 0.6 mm for female subjects aged between 26 and 45 years old. On the other hand, Harris,^25^ after conducting a longitudinal assessment of individuals aged between 20 and 54 years old, did not find changes in intercanine width. Richardson and Gormley,^22^ after a 10-year follow-up of adults aged between 18 and 28 years old, found that changes in intercanine width were minor.

The present study corroborates the decrease in lower dental arch length during childhood,^4^^,^ 7 ^,^ 8 ^,^ 25 ^,^ 27 adolescence,^4^^,^ 7 ^,^ 23 and adulthood^3^^,^ 6 ^,^ 22 reported in previous studies (Table 1). Total reduction in arch length was 1.28 mm, 0.51 mm on the right side (p < 0.001) and 0.77 mm on the left side (p < 0.001).

Intermolar width showed a statistically significant (p = 0.0022) average decrease of 0.43 mm (Table 1). This is the most controversial measurement in the literature, particularly because while some studies found no significant differences,^1^^,^ 3 ^,^ 4 ^,^ 7 ^,^ 23 ^,^ 24 others observed an increase in intermolar width when assessing adolescents and young adults.^6^^,^ 22 ^,^ 25 This divergence is probably due to methodological differences, especially with regard to the age range of research subjects.

In this paper, regression analysis revealed that increased crowding in the third decade of life could not be predicted by any variable examined at the end of the second decade, neither based on arch dimensions nor Little’s index. Similar findings have been reported for the upper arch in an adolescent sample.

Another study presented untreated subjects who had higher or lower degree of dental crowding in the lower arch in a first evaluation, and showed similar findings in the second evaluation. Assessments were carried between the age of 15 and 20 years;^20^ therefore, severity of initial irregularity would not cause the subject to have a worse prognosis of crowding in the future. However, a longer evaluation period is required to confirm the results.

Dental crowding is a progressive feature of aging. Examining untreated subjects is key to understand the changes that occur during natural aging. Physiological changes in the morphology of dental arches seem to occur throughout life, but our results suggest that these changes are milder after the second decade of life. Scientific evidence is required to identify which factors actually influence this process.

## CONCLUSIONS

Lower dental arch dimensions tend to minor, but significant, decrease from 20 to 30 years of age, whereas dental crowding increases in an average of 1 mm during this period.

Changes in dental alignment in the third decade of life cannot be predicted on the basis of arch dimensions or the severity of dental crowding at the end of the second decade of life.
